# The effect of contest participation and contest outcome on subsequent prosocial behavior

**DOI:** 10.1371/journal.pone.0240712

**Published:** 2020-11-03

**Authors:** Adiel Moyal, Ilana Ritov

**Affiliations:** 1 Department of Cognitive Sciences, The Hebrew University of Jerusalem, Jerusalem, Israel; 2 School of Education and Center for Rationality, The Hebrew University of Jerusalem, Jerusalem, Israel; Middlesex University, UNITED KINGDOM

## Abstract

Following previous research on various aspects of contests, we aim to explore how taking part in a contest affects subsequent behavior. We focus on whether the experience of having just competed in a contest, beyond its outcome, would have an impact on other-regarding decisions towards an individual who was not part of the preliminary contest. In addition, in light of inconclusive results in the existing literature regarding the effect of contest outcome on subsequent prosociality, we reexamine this effect. In line with our hypothesis, participation in a contest was found to reduce prosociality. Additionally, we found that winning a contest reduced prosociality only when decisions were framed as “giving” decisions and not as “dividing” decisions. This finding suggests that the effect of contest outcome may depend on specific elements of the presented situations.

## Introduction

Individuals have engaged in contests and competitions throughout history. Nowadays, interpersonal competitions are present in all life arenas, and may take different forms that are influenced by individuals and situational variables [1]. In fact, competitions are not only present, but also serve as a situational and potentially influential factor within central spheres of life, such as work and education [2]. Therefore, there is much interest in investigating the impacts of a preliminary contest on varied types of prosocial behaviors. The current work aims to explore the effect of participation in an earlier contest, beyond a specific outcome, on prosocial behavior in a subsequent, independent situation, expanding the body of research on contest impact. In addition, due to inconsistent findings regarding the effect of contest outcome on subsequent prosociality, we will reexamine this effect.

Competitive situations have psychological, physiological and behavioral influences [3, 4]. Among other findings, competitive environments were found to encourage unethical behavior in the lab, in the context of kickbacks [5], and out of the lab, in the form of tax avoidance [6]. In addition, within the interaction of a sequential 10-round dictator game, Duffy & Kornienko [7] found that ranking participants according to how high their allocations were relative to other dictators' allocations reduced generosity.

Prosocial behavior has been linked to empathy (e.g., [8–12]), which is an emotional response that focuses on others [13]. For instance, high empathy levels were found to induce increased altruistic behavior that was expressed by greater resource allocation to the target of the empathy [14, 15].

Engaging in competitive contests may be associated with reduced empathy. Zaki [16] used the illustration of a linebacker being unable to do his job well if he shares the pain of players he tackles as an example of the fact that empathy may interfere with performance in competition. Cikara, Bruneau, Van Bavel & Saxe [17] investigated the effect of competition on empathy and found that competition between groups reduced empathy and increased individuals’ counter-empathic responses toward out-group members. Along the same lines, Lanzetta & Englis [18] demonstrated that expectations of competition resulted in counter-empathetic physiological expressions.

Since competitive environments are associated with reduced empathy, which is in turn related to reduced prosociality, and in light of the presented evidence demonstrating reduced prosociality in the competitive interaction itself (e.g., [7, 19]), we hypothesize that participation in a competitive contest, beyond the contest outcome, will reduce prosociality in a subsequent, unrelated situation.

In addition to the hypothesized effect of participation in a contest on subsequent prosocial behavior, we are also interested in examining the effect of contest outcome–winning versus losing–on this behavior. Intuitively and theoretically, there are reasons to believe that winners will be more prosocial than losers, as well as reasons to predict that losers will be more prosocial than winners. On the one hand, and as mentioned by Kidd, Nicholas & Rai [20], according to the warm glow theory [21] winners are anticipated to be more prosocial because they are in a good mood in light of their recent win. On the other hand, Kidd et al. [20] also mentioned that according to the negative state-relief model [22] losers are predicted to be more prosocial as an attempt to improve their bad mood that was generated by the loss. These two possible and contradictory predictions are based on findings that winning or losing a contest accordingly evokes positive or negative emotions (e.g., [23, 24]), and that good feelings (e.g., [25]) as well as bad feelings (e.g., [26]) increase prosociality.

In addition to the theoretical basis for the hypotheses predicting higher prosociality for both contest winners and losers, the experimental findings from earlier works are inconclusive. In a work by Kidd et al. [20], individuals’ donations to charity were measured after a contest between four participants, and it was found that winners were more generous than the others. In contrast, in a work by Schurr & Ritov [27], winners tended to cheat more than losers did in order to maximize their monetary profit at the expense of other participants. However, there are two main differences between these two above-mentioned works. Whereas in the work by Kidd et al. [20] winners earned more money than the others did due to their victory, in the work by Schurr & Ritov [27] winners did not earn more money than losers, although they were rewarded with a pair of earbuds. Another difference, as previously mentioned, is the number of opponents in the contest. While in the Kidd et al. [20] experiment there were four participants in the contest, in the Schurr & Ritov [27] experiment there were two participants in each contest. I.e., each participant was either a winner or a loser. These two differences lead us to hypothesize that in a contest between two opponents, in which the prize for winning may not be used as a resource in a subsequent decision, winners will be less prosocial than losers.

This hypothesis is supported by two lines of research, one which suggests that winners are akin to high-status group members, and another that has linked high status to reduced prosocial behavior. The first line of research refers to winning or losing a contest as a respective upward or downward shift in status (e.g., [23, 28]), and therefore suggests that winning a contest may serve as a high-status marker [29]. The second line of research demonstrates that high-status individuals are less tolerant of resource distribution in which their portion is significantly smaller than the other party’s [30, 31]. Furthermore, high status is related to increased unethical behavior, such as approving unethical actions [32] or actually cheating in order to increase one’s chances to win a prize [33]. Finally, and highly relevant to our work, high status has been linked to reduced prosociality (as reviewed by Piff & Robinson [34]), such as in resource allocation decisions [35] and in helping behavior [36].

Based on the findings that high status is related to an increased sense of entitlement [37], and that entitlement is negatively related to several aspects of prosociality [38], the decreased prosociality of high-status members has been linked to increased entitlement [34]. In addition, it was found that equality priming eliminated entitlement differences between high and low-status members [37]. Therefore, it makes sense to assume that the salience of equality concerns would also diminish differences in prosociality between the two groups. Support for this reasoning is provided by a study demonstrating that dictators' allocation decisions were dependent on their family income and decision framing [39]. The researchers found that whereas high-income dictators allocated less to the other when the decision was framed in terms of giving, this difference was not found when the decision was framed in terms of dividing.

The similarity between winners and high-status members, and the finding of Schurr & Ritov [27] that winning a contest enhances a sense of entitlement, lead us to explore whether framing the task such that it evokes equality considerations would diminish, or even eliminate, the negative effect of winning a contest on prosociality in subsequent situations. Indeed, task framing may serve as a useful tool through which policy makers can affect decisions without investing further incentives (e.g. choice architecture [40, 41]; decisions framing, [39, 42–44]).

## The current work

The current work is based on four experiments that each consisted of two main stages. The first stage was participation in a game, which was presented to participants in the treatment groups (i.e. winners and losers) as competitive, and to control group participants as having no competitive elements. The second stage consisted of making a decision that served as a measure of participant prosociality. For the sake of generality, we implemented two behavioral measurements of prosocial behavior–resource allocation and labor allocation.

In Experiment 1 we demonstrated the effect of taking part in a contest, and of contest outcome, on subsequent prosocial behavior measured through a resource allocation decision–a hypothetical dictator game. Experiments 2.1 and 2.2 were conducted in order to eliminate alternative explanations for the results obtained in Experiment 1. Experiment 3 replicated the findings of Experiment 1 using a real money dictator game as the dependent variable. In Experiment 4 we aimed to enable a broader generalization of our previous findings by using a labor allocation decision as the dependent variable. Additionally, in this final experiment we demonstrated the interaction effect between the outcome of a contest and the framing of the decision. [The data files can be found at *https://osf.io/mxj76/?view_only=aced4d03ba1a46abac9c0375f55e44a6*]

## Experiment 1

Allocation of resources in a dictator game served as the current work’s first dependent variable. For Experiment 1 we used a hypothetical version of the dictator game and we hypothesized that: (H1) Participants who took part in a contest would allocate less money than participants who did not take part in a contest to their partner in the dictator game. (H2) Participants who won the contest would allocate less money than participants who lost the contest to their partner in the dictator game.

### Method

#### Participants

The participants (*N* = 357) were Mechanical Turk (MTurk) workers who were invited to take part in the experiment in exchange for 1 U.S dollar. Their mean age was 33.18 (*SD* = 9.88) and 44.5% of them were women. The participants were randomly assigned to one of the three groups: Winners (*N* = 116), Losers (*N* = 118) or Control (*N* = 123). A sensitivity power analysis was conducted with G*power [45]. The result indicated that given the current sample size, with α = .05 and power of 0.8, statistical significance would be detected with small to medium effect size (Cohen's *f* = 0.16).

#### Procedure

The experiment Ethics approval was provided by the School of Education at the Hebrew University of Jerusalem (2020Y1208), which was administered online, consisted of 2 main stages and the recording of demographic information. In the first stage, participants completed a task. The task was presented as a competitive game for participants in the treatment groups (i.e. winners and losers), and simply as a game for participants in the control group. I.e., whereas participants in the treatment groups were ostensibly matched into pairs of opponents, and were told that the winner would participate in a $5 raffle, participants in the control group completed exactly the same task, but without any competitive elements (See S1 Appendix for a detailed description).

In the treatment groups, after completing the task participants received a notification that they had won or lost the game. (Although the message ostensibly referred to their performance, in effect it was randomly determined). The winning notification informed winners that they would be included in the raffle. Immediately after receiving this notification, participants were informed that this section of the experiment had ended, and that they would be transferred to another screen unrelated to the task they had just completed. Participants in the control group received this 'transfer message' right after completing the task.

In the second stage of the experiment, all of the participants took part in a hypothetical dictator game, as the dictators. The participants were asked to imagine that they had received an endowment of $10, and then to indicate how much of the endowment they would allocate to another participant. Decisions could be any amount from $0 to $10, in increments of $1. Finally, the participants recorded their age and gender.

### Results

#### Ability and effort put into the task

First, we wanted to verify that any difference found between the groups could not be explained by participants having different abilities or having invested different amounts of effort into the game. Participants had a fixed time to answer each question in the game, which was competitive for the treatment groups. Therefore, the ability and amount of effort put into the game was measured by the number of correctly answered questions, out of a total of sixteen questions. The relevant analyses revealed that participants in the treatment groups (*M* = 10.51 *SD* = 2.66) did not perform better than participants in the control group (*M* = 10.31 *SD* = 2.52), *t*(355) = 0.69, *p* = .49.

#### Decisions in the dictator game

A one-way ANOVA showed a significant effect of treatment group on mean giving *F*(2,354) = 5.93, *p* = .003, Cohen's *f* = 0.18. Planned contrasts revealed the following: (1) The participants from the two contest groups, taken together, gave less (*M* = $2.67, *SD* = 2.29) than the control group (*M* = $3.36, *SD* = 2.12), *t*(354) = -2.78, *p* = .006, Cohen's *d* = 0.31. (2) The winners gave significantly less (*M* = $2.37, *SD* = 2.19) than the losers (*M* = $2.97, *SD* = 2.36), *t*(354) = 2.04, *p* = .04, Cohen's *d* = 0.26. (3) Whereas winners gave significantly less than the control group, *t*(354) = -3.42, *p* = .001, Cohen's *d* = 0.46), losers did not significantly differ from the control, *t*(354) = -1.36, *p* = .17, Cohen's *d* = 0.17. The demographic variables did not interact with the independent variable.

### Discussion

In line with our prediction, participating in a preliminary contest had a negative effect on prosocial behavior, as expressed in hypothetical resource allocation decisions. This effect seems to be driven mainly by the winners. Interestingly, winners were less generous than losers, in keeping with Schurr & Ritov’s [27] finding that winners demonstrated more unethical behavior than losers, which may represent another aspect of prosocial behavior. However, this result seems to be different from the finding of Kidd et al. [20], who found that winners donated more than others.

## Experiment 2.1

In light of the procedure and results reported in Experiment 1, it could be argued that the possibility of winning a prize, rather than the engagement in a contest, led to the reduced prosociality observed in the subsequent interaction. Similarly, the reduced prosociality that we attributed to winning a contest could alternatively be attributed to winning a prize. In order to test these alternative explanations, we designed and conducted Experiment 2.1.

Experiment 2.1 included three groups: two groups that were equivalent to the treatment groups from Experiment 1, and a control group. Participants in the treatment groups had a chance to win participation in a raffle for a $5 Amazon Gift Card without engaging in a contest, while participants in the control group did not have that chance. If the effects in Experiment 1 indeed stemmed from the chance to participate in a raffle, then this experiment should yield equivalent results. I.e., we would expect to find a significant difference between participants who had the chance to win raffle participation (treatment groups) and participants who did not (control group). In addition, a significant difference would be expected between participants who won the raffle participation ("random winners") and participants who had the chance to win but did not ("random losers"). In contrast, if this experiment does not yield significant results, it would eliminate the alternative explanations and would therefore support our conclusion regarding the effect of preliminary contest and its outcome on subsequent prosocial behavior.

### Method

#### Participants

The participants (*N* = 363) were MTurk workers who were invited to take part in the experiment in exchange for 0.15 U.S dollars. The mean age of the participants was 35.56 (*SD* = 11.17) and 51% of them were women. The participants were randomly assigned to one of the three groups: (1) Random Winners (*N* = 119), in which participants learned that they may (randomly) win participation in a raffle, and were then informed that they would participate in the raffle. (2) Random Losers (*N =* 122), in which participants learned that they may (randomly) win participation in a raffle, and were then informed that they would not participate in the raffle. (3) Control group (*N* = 122), in which no raffle was mentioned and no participant won participation in a raffle. A sensitivity power analysis was conducted with G*power [45]. The result indicated that given the current sample size, with α = .05 and power of 0.8, statistical significance would be detected with small to medium effect size (Cohen's *f* = 0.16).

#### Procedure

In order to maintain similar procedure to that of Experiment 1, the participants from the treatment groups were informed that they were randomly matched into pairs and that one participant from each pair would be randomly determined to participate in a raffle for a $5 Amazon Gift Card. These participants received a number (e.g. "Your number for this task is Participant number 118"), and their matched participant was also identified by a number. After that, half of these participants were informed that they had been randomly determined to participate in the raffle, and the other half were informed that it was randomly determined that they would not participate in the raffle. All participants were then directed to a dictator game, described below. Participants in the control group did not receive any information regarding a raffle, and directly faced the dictator game.

Next, participants read that they were to be paired with another participant who would be referred to as their counterpart for this stage. Participants from the treatment groups were informed that the counterpart could not be the same individual from the previous stage. Then the participants took part in a dictator game allocating $10, as dictators. The instructions indicated that some of them would receive a bonus payment according to their decisions, and were therefore asked to make a decision that reflects their real preferences. The participants then indicated how much of an initial endowment of $10 they decided to allocate to the counterpart, from $0 to $10, in increments of $1. Finally, the participants recorded their age and gender.

#### Payment method

The payment of the participants who were randomly determined to receive a bonus according to the decisions made in the dictator game consisted of two components: (1) the basic payment and (2) a bonus payment according to the decision in the dictator game.

### Results

A one-way ANOVA showed no significant effect of treatment group on mean giving *F*(2,360) = 0.17, *p* = .85. Planned contrasts did not reveal any significant effects either: (1) The participants from the two treatment groups, taken together, did not give a significantly different amount (*M* = $3.4, *SD* = 2.31) than the control group (*M* = $3.25, *SD* = 2.29), *t*(360) = 0.56, *p* = .57. (2) Among the treatment groups, random winners did not give a significantly different amount (*M* = $3.38, *SD* = 2.43) than random losers (*M* = $3.42, *SD* = 2.21), *t*(360) = 0.13, *p* = .89. The demographic variables did not interact with the independent variable.

### Discussion

There were no significant effects observed in this experiment, in contrast to the prediction of the alternative explanation, which attributes subsequent prosocial behavior to the possibility of winning a prize and not to participation in a contest. The absence of significant differences in the levels of prosociality between the treatment groups and the control group, as well as between participants who won participation in a raffle and those who did not, strengthens our argument that participation in a contest, and contest outcome, impact subsequent prosocial decisions, and negates the possibility of winning a prize as the source of Experiment 1’s results.

## Experiment 2.2

Another alternative explanation for the negative effect of winning a contest on prosociality may be related to anticipated emotions. Specifically, it could be argued that participation in a future raffle, which was the prize of winning the contest, led the contest winner to envision a possibility in which they may not win the raffle and would therefore be left with no tangible prize at all. This insight may create negative feelings such as disappointment or frustration, which may in turn lead to reduced prosociality.

In order to test this alternative explanation, Experiment 2.2 directly probes the anticipated emotions of winners pertaining to the raffle outcomes. As in Experiment 1, participants engaged in a contest in which winners won participation in a raffle for a $5 Amazon Gift Card. If the reduced prosociality of winners in Experiment 1 was a result of anticipated disappointment due to the possibility of not winning the future raffle, it would be expected that when winners think of their winning and of the possible outcomes of the future raffle, it would evoke negative emotions. In contrast, if winners report overall high positive emotion, we can conclude that although they have not obtained the outcome of the raffle, the anticipated participation in the raffle evokes positive emotions, and this would not support the role of anticipated disappointment as the source of the effect.

### Method

#### Participants

The participants (*N* = 222) were MTurk workers who were invited to take part in the experiment in exchange for 0.50 U.S dollars. The mean age of the participants was 37.25 (*SD* = 12.91) and 50% of them were women. The participants were randomly assigned to one of the two groups: Winners (*N* = 109) or Losers (*N =* 113). A sensitivity power analysis was conducted with G*power [45]. The result indicated that given the current sample size, with α = .05 and power of 0.8, statistical significance would be detected with small to medium effect size (Cohen's *d* = 0.33).

#### Procedure

This experiment was administered online, in keeping with the previous experiments. Initially, participants took part in the same game as the participants in Experiment 1, with participation in a $5 Amazon Gift Card raffle as incentive to win the game. After completing the game, participants received a random notification that they either won or lost the game, including the information that due to their win or loss they will (winners) or will not (losers) participate in the $5 Amazon Gift Card raffle. Next, participants were asked to report how they felt in regard to their contest outcome and its implications. Specifically, winners read the following instructions: “Considering your victory along with the probability that you will not win the gift card raffle and the probability that you will win the gift card raffle, please indicate to what extent you feel each of the following…”. Conversely, losers read the following instructions: “Considering your loss and that you will not participate in the gift card raffle, please indicate to what extent you feel each of the following…”

Then, all participants indicated to what extent they felt disappointed/elated, unhappy/happy and frustrated/satisfied, on 11-point scales ranging from ‘-5’ (very disappointed/unhappy/frustrated) to ‘+5’ (very elated/happy/satisfied), with ‘0’ labeled as “neutral”. Finally, the participants recorded their age and gender.

### Results

The results indicated that winners reported positive emotions rather than negative or neutral emotions, despite being prompted to think of the possibility of either winning or not winning the raffle. T-test analyses yielded that (a) winners reported higher levels of elation (*M* = +2.83, *SD* = 1.94) than the neutral point, *t*(108) = 15.28, *p* < .001; (b) winners reported higher levels of happiness (*M* = +3.24, *SD* = 1.6) than the neutral point, *t*(108) = 21.08, *p* < .001; and (c) winners reported higher levels of satisfaction (*M* = +3.06, *SD* = 1.88) than the neutral point, *t*(108) = 17, *p* < .001). Conversely, losers reported negative emotion on each of the three scales (*p* < .001).

In addition, results show that winners reported higher levels of positive emotions in comparison to losers, even though they were asked to think about the possibility of not winning the raffle. On the same scales reported above, t-test analyses yielded that winners reported (a) higher levels of elation (*M* = +2.83, *SD* = 1.94) than losers (*M* = -2.04, *SD* = 2.04), *t*(220) = 18.26, *p* < .001; (b) higher levels of happiness (*M* = +3.24, *SD* = 1.6) than losers (*M* = -1.89, *SD* = 1.95), *t*(220) = 21.39, *p* < .001; and (c) higher levels of satisfaction (*M* = +3.06, *SD* = 1.88) than losers (*M* = -1.72, *SD* = 2.03), *t*(220) = 18.17, *p* < .001).

### Discussion

The results of this experiment do not support the alternative explanation that the observed difference between winners’ and losers’ prosociality results from negative feelings evoked in winners because their prize was participation in a raffle rather than a sure gain.

## Experiment 3

The present experiment sought to replicate the findings of the effect of contest and its outcome on prosocial behavior from Experiment 1. This replication is important because, as mentioned in the introduction, there were theoretical and empirical findings that would have predicted the opposite results of those observed in Experiment 1. In addition to the replication itself, this experiment aims to examine whether the effects found in Experiment 1 would replicate when decisions have real monetary consequences.

According to the findings from Experiment 1 and the reviewed literature, we hypothesized that: (H1) Participants who took part in a contest would allocate less money in the dictator game, compared to participants who did not take part in a contest. (H2) Winners would allocate less than losers in the dictator game.

### Method

#### Participants

The participants (*N* = 369) were MTurk workers who were invited to take part in the experiment in exchange for 1 U.S dollar. Their mean age was 33.98 (*SD* = 9.96) and 42.5% of them were women. The participants were randomly assigned to one of the three groups: Winners (*N* = 124), Losers (*N* = 122) or Control (*N* = 123). A sensitivity power analysis was conducted with G*power [45]. The result indicated that given the current sample size, with α = .05 and power of 0.8, statistical significance would be detected with small to medium effect size (Cohen's *f* = 0.2).

#### Procedure

The experiment consisted of two main stages, and the entirety of the experiment was administered online. In the first stage, participants took part in the same game as the participants in Experiment 1, with participation in a $5 Starbucks Gift Card raffle as incentive for participants in the treatment group to win the game.

After completing the game, participants in the treatment groups responded to competition check questions. The participants indicated, on a scale between 0 to 10: (1) To what extent they wanted to win the game (0 = Do not want to win at all; 10 = Greatly want to win). (2) To what extent they believed that the game reflected ability over luck (0 = The game reflected mostly luck; 10 = The game reflected mostly ability). (3) To what extent they wanted not to lose the game (0 = Do not want to lose at all; 10 = Do not care if I lose or not). Then, the participants received a random notification that they had won or lost the game. Immediately after receiving this notification, the participants were informed that this section of the experiment had ended, and that they would be transferred to another screen unrelated to the task they had just completed.

As in Experiment 1, the participants from the control group completed exactly the same task as the participants from the treatment groups, but without any elements of competition, and were then transferred to the next section.

For the second stage of the experiment, all of the participants took part in a standard dictator game, as the dictators. The instructions explained that each decision maker would receive $1 and that they should decide how much of it to keep for themselves and how much to give to the recipient. The decision maker could divide this amount in increments of ten cents.

Important to note is the emphasis placed on explicitly expressing to participants that the recipient of the dictator game was not the participant against whom they had competed in the first stage of the experiment. This distinction allowed us to eliminate alternative motivations regarding the competitive history between the decision maker and the target of his decisions as explanations for any observed effects. Finally, the participants recorded their age and gender.

#### Payment method

Each participant’s payment consisted of three components: (1) the basic payment for participating in the experiment (1 U.S dollar). (2) A bonus payment according to their decision in the dictator game (i.e. a bonus stemming from their role as dictators). (3) A bonus payment according to the decision of another participant, to whom they were randomly paired to as recipients (i.e. a bonus stemming from their role as recipients). It is important to note that the participants were not informed, either in advance or during the experiment, that they would play the role of recipient in addition to their role as dictator. I.e., participants received an additional bonus according to their role as recipients, but they were not informed of the origin of this bonus.

### Results

#### Ability and effort put into the task

As in Experiment 1, we compared the number of correct questions in both groups and found that participants in the treatment groups did not perform (*M* = 9.89, *SD* = 2.71) significantly differently than participants in the control group (*M* = 9.6, *SD* = 2.48), *t*(367) = 0.99, *p* = .32.

#### The competition check

The competition check questions, which participants in the treatment groups responded to, showed that they wanted to win the game (*M* = 7.2, *SD* = 2.45), believed that the game reflected more ability than luck (*M* = 7.22, *SD* = 2.53), and that they wanted not to lose the game (*M* = 2.85, *SD* = 2.98). The significant negative correlation between the willingness to win and the desire not to lose (*r* = -0.6, *p* < 0.001) indicates that participants did not respond randomly to these questions.

#### Decisions in the dictator game

In general, the results replicated the findings of Experiment 1. A one-way ANOVA showed a significant effect of treatment group on mean resource allocation *F*(2,366) = 3.71, *p* = .03, Cohen's *f* = 0.14. Planned contrasts revealed the following: (1) Participants from the two contest groups, taken together, allocated lower amounts of money (*M* = ¢16.91, *SD* = 23.94) to the recipients compared to the control group (*M* = ¢22.03, *SD* = 22.97), *t*(366) = -1.96, *p* = .05, Cohen's *d* = 0.22. (2) Winners allocated marginally less to their recipients (*M* = ¢14.11, *SD* = 21.75) than did the losers (*M* = ¢19.75, *SD* = 25.76), *t*(366) = 1.88, *p* = .06, Cohen's *d* = 0.24. (3) Whereas winners gave significantly less than the control group, *t*(366) = -2.64, *p* = .01, Cohen's *d* = 0.35), losers did not give significantly different amounts from the control group, *t*(366) = -0.76, *p* = .45, Cohen's *d* = 0.09. The demographic variables did not interact with the independent variable.

### Discussion

This experiment provided further support for the negative effect of contest on prosocial behavior. In line with our prediction, and with the results from the hypothetical dictator game in Experiment 1, participating in a preliminary contest reduced prosocial behavior as expressed in resource allocation decisions; an effect that seems to be driven solely by the winners. Additionally, the finding that winners seem to be less generous than losers replicated both when the allocations were hypothetical and when they were made using real money.

## Experiment 4

After finding and replicating the effect of a preliminary contest and its outcome on prosociality, measured by subsequent resource allocation decisions, we wanted to examine whether the negative effect of winning a contest on subsequent prosociality would replicate in another aspect of prosocial behavior that is highly relevant for organizational and managerial contexts–labor allocation. In addition, we wanted to explore the robustness of the negative effect of contest outcome when the decision is presented in a variety of ways.

In light of the previously mentioned finding, according to which equality priming may diminish differences between high- and low-status members [37], we wanted to explore whether such priming would eliminate the negative effect of winning a contest on prosociality. In order to do that, we added another independent variable: the framing of the decision. In one condition the decision was framed as a 'dividing' decision, and in the other condition the decision was framed as a 'giving' decision. Intuitively, and as research suggests [39], we expected equality concerns to be raised as a result of the 'dividing' framing.

Thus, in addition to our hypothesis that in the giving framing winners would allocate more labor to others compared to losers, we wanted to test whether the difference between the two groups would disappear in the dividing framing.

### Method

#### Participants

The participants (*N* = 440) were MTurk workers who were invited to take part in the experiment in exchange for 0.5 U.S dollars. Their mean age was 37.64 (*SD* = 12.27) and 52.7% of them were women (thirteen participants did not report their gender). A sensitivity power analysis was conducted with G*power [45]. The result indicated that given the current sample size, with α = .05 and power of 0.8, statistical significance would be detected with small to medium effect size (Cohen's *f* = 0.16).

#### Procedure

The design was a 2 (contest outcome) X 2 (framing) between-subject design. The entirety of the experiment consisted of two main stages, administered online. In the first stage, participants took part in the same contest as in Experiments 1, 2.2 and 3, in which a $5 Amazon Gift Card raffle served as an incentive to win. After completing the game stage, participants were asked to answer the competition check questions. The participants then received a random notification that they had won or lost the game. Immediately after receiving this notification, the participants were informed that this section of the experiment had ended, and that they would be transferred to another screen unrelated to the task they had just completed.

In the second stage of the experiment, the participants read that they were to be paired with another participant (not the opponent from Stage 1) who would be referred to as their counterpart. Subsequently, participants saw an example of a "translation question”: "*What is the Finnish translations of the Polish word 'Dobry'*?*–zły / łóżko / czarny / hyvä / There is no correct answer available*". Then, participants faced the labor allocation decision.

In the dividing framing, participants were informed that each pair had to answer ten "translation questions" together, and that they had been randomly selected to determine how many questions each of the participants in the pair would answer. In the giving framing, participants were informed that their counterpart had been randomly selected to answer ten "translation questions", and that they could give help and answer some of the questions instead of their counterpart. I.e. each question answered by the decision makers would be reduced from the total ten questions that their counterpart had to answer. Then, in both conditions, participants chose how many questions to answer by themselves, out of the total ten. Finally, the participants recorded their age and gender.

### Results

#### The competition check

The competition check questions, to which participants in the treatment groups responded, showed that they wanted to win the game (*M* = 7.44, *SD* = 2.28), believed that the game reflected more ability than luck (*M* = 7.78, *SD* = 2.26), and that they wanted not to lose the game (*M* = 3.02, *SD* = 2.9). The significant negative correlation between the willingness to win and the desire not to lose (*r* = -0.44, *p* < 0.001), indicates that participants did not respond randomly to these questions.

#### Labor allocation decisions

**Fig 1:**
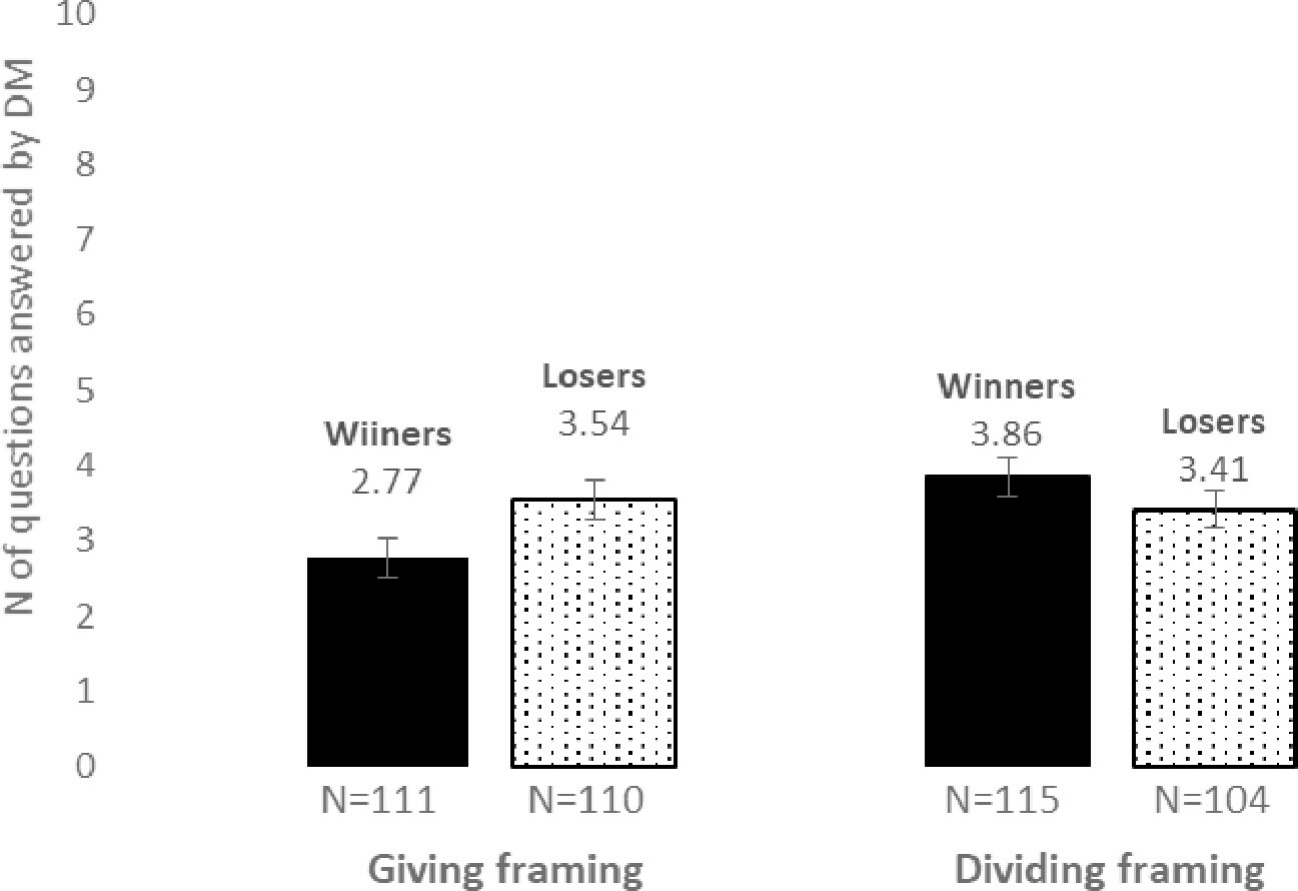
Mean number of questions that participants answer by themselves as dependent on treatment group. Error bars represent one standard error.

The mean number of questions that the participants from each of the groups chose to answer by themselves is displayed in Fig 1.

A two-way ANOVA demonstrated no significant effect of contest outcome *F*(1,436) = 0.37, *p* = .54, Partial *η*2 = 0.001, a nearly significant effect of framing *F*(1,436) = 3.34, *p* = .07, Partial *η*2 = 0.008, and a significant interaction effect *F*(1,436) = 5.24, *p* = .02, Partial *η*2 = 0.012. These analyses indicate that winners did not answer a significantly different number of questions than the losers, and that participants in the giving framing answered nearly significantly fewer questions than participants in the dividing framing. In addition, the analyses indicate that the difference between winners and losers varied depending on the framing. Follow up t-test analyses for each framing separately showed that whereas there was no significant difference between the number of questions that winners (*M* = 3.86, *SD* = 2.83) and losers (*M* = 3.41, *SD* = 2.57) answered in the dividing framing, *t*(217) = 1.22, *p* = .22), this was not the case in the giving framing. In the giving framing, winners (*M* = 2.77, *SD* = 2.86) answered significantly fewer questions than losers (*M* = 3.54, *SD* = 2.87), *t*(219) = -2, *p* = .047, Cohen's *d* = 0.27.

### Discussion

The significant difference between winners and losers in the giving framing can be interpreted as a conceptual replication of our findings from previous experiments. In other words, we have again found that winners were less prosocial than losers. In addition, this effect was not limited to monetary decisions, but was also found to exist when prosociality was measured by the time and effort that individuals dedicated to helping one another. We note that this finding cannot be accounted for simply by assuming that winners anticipate being disappointed by not winning the gift card.

## General discussion

The current work makes two major contributions to the large body of research on contests, and particularly to the growing line of research that investigates influences of preliminary contests and their outcomes on subsequent decision-making (e.g., [19, 20, 27, 46]). The first contribution is the presentation of the robust effect of contest participation on prosocial behavior in subsequent situations. The second contribution is the finding that the effect of winning a contest on prosociality is frame-dependent. I.e., winning a contest harms prosociality only when the decision is framed as a “giving” decision, but not when it is framed as a “dividing” decision.

The effect of contest participation on subsequent prosociality suggests that the effect found by Duffy & Kornienko [7], according to which generosity is reduced due to a competitive interaction, is not limited to the specific interaction involving the contest, but may spill over to other situations. Notably, this effect may be related to rivalry formation [47] and to the link between rivalry and unethical behavior [48, 49].

Interestingly, our results are also in line with the findings by Peysakhovich & Rand [50], according to which competitive strategies that were adopted during a preliminary interaction due to its environmental structure were carried over and were expressed later through less prosocial decisions. The authors [50] explained their results in light of the social heuristics hypothesis [51]. Specifically, they suggested that the early interactions, wherein competitive strategies were dominant, made a less prosocial decision the intuitive behavior in the later interaction. Note that although the social heuristics hypothesis was initially presented in the context of cooperation, it was later demonstrated in the context of altruism [52] and was also formalized in a general model [53]. In fact, our findings that preliminary contest harms prosociality could be interpreted along the same lines. I.e., competitive behavior becomes the default behavior during the contest stage, and this competitive strategy then spills over to the subsequent decision situation, although there is no reason to connect the two independent situations.

The frame-dependent effect of winning suggests that winners are predicted to be less prosocial than losers in decisions that involve giving of oneself to benefit others. In addition, this effect may suggest that the influence of contest outcome interacts with other factors present in the decision-making environment. Specifically, we showed that the effect of contest outcome on prosociality depended on the perceived role of the decision maker–as a potential giver vs. divider. More broadly, and based on the literature that linked contest outcomes to perceived status (e.g., [23, 28, 29]), this finding hints that the effect of social status on prosocial behavior may depend on the way the decision makers’ perceive their role. Finding that the negative effect on prosociality holds only in a giving framing and not in a dividing framing is important not only from a theoretical perspective, but also because it may help enhance desirable behavior and inhibit undesirable behavior.

Referring to the earlier discussed discrepancy between findings by Kidd et al. [20] and Schurr & Ritov [27] regarding the effect of winning on subsequent prosocial decisions, our findings are in line with the results of Schurr & Ritov [27]. The contradictory results found by Kidd et al. [20], that winners donated to charity more than the other competitors, may be explained by the fact that their winners had earned more money than the others–due to their victory–and were then asked to decide how much of their earnings to donate. In other words, not only was the preliminary contest connected to the donation decision by the fact that the participants’ initial endowments were determined by their contest’s outcome, but the decision instructions also explicitly mentioned that the donations would come from the participant’s earnings. In this context, it is important to mention that although Kidd et al. [20] controlled for the absolute earning between winners and losers, they did not control for the relative earning of winners in comparison to the other competitors. Hence, this design did not completely isolate the preliminary contest from the subsequent donation decision. Specifically, the effect of winning a contest was not disentangled from the effect of receiving a higher reward than others. According to the social comparison literature, receiving a higher (lower) reward than others places the individual in a situation of downward (upward) social comparison. Therefore, research showing that upward social comparison inhibits prosocial behavior (e.g., [54]) while downward social comparison enhances prosocial behavior (e.g., [55, 56]) may explain the results obtained by Kidd et al. [20].

In fact, the different results obtained by Kidd et al. [20] and Schurr & Ritov [27], together with our finding regarding the interaction between contest outcome and perceived role, demonstrate the complexity of contest outcome’s effect on prosocial behavior. I.e., the effect of contest outcome on prosociality may be particularly context-sensitive, in more than one sense. Specifically, in addition to contest characteristics and prize methods, the effect of contest outcome on prosociality is mediated and/or moderated by emotions and thoughts evoked by the preliminary contest and perhaps related to the contest design. Interestingly, interpersonal differences may also act as mediators and moderators. Thus, future work could focus on additional characteristics of the decision-task or the contest environment, which may interact with the effect of contest outcome on prosocial behavior, and on other types of behaviors. In addition, further research may focus more on the possible interpersonal differences with regard to the relevant effects.

The current work also has organizational and managerial implications. For example, in regards to organizational methods that aim to increase employees' productivity, our findings suggest that negative side effects should be considered. First, the extent to which such methods are employed should be monitored. Second, actions should be taken in order to prevent subsequent antisocial behavior after social comparisons. In this context, relevant field experiments may be extremely useful.

## Supporting information

S1 Appendix A detailed description of the (competitive) game. (DOCX)
