# Supplementary material for: The effect of contest participation and contest outcome on subsequent prosocial behavior
Source: PLoS One. 2020 Nov 3;15(11):e0240712. doi: 10.1371/journal.pone.0240712 (PMC7608921; doi:10.1371/journal.pone.0240712)
Supplement: S1 Appendix — (DOCX) [file pone.0240712.s001.docx]

**The effect of contest participation and contest outcome on subsequent prosocial behavior**

**S1 Appendix: A detailed description of the (competitive) game**

The design of the (competitive) game

One topic that has been examined in the context of contests is that of variables which influence the degree of competitiveness (e.g., [57-61]). For instance, Garcia, Tor, & Gonzales [57] investigated variables of the competitive situation itself, and found that competitiveness increases when competitors’ ranks are close to the extremes of the scale, i.e. in cases in which the competitors are close to the winning position, or alternatively, to the last, losing position. This finding suggests that in contest between only two contestants the degree of competitiveness would be high. The participants in this study were therefore informed that they would compete against only one other participant, meaning that each participant would be either the winner or the loser. Moreover, work by Haran & Ritov [59] demonstrated that the identifiability of one’s opponent increases the degree of competitiveness. Therefore, in our experiments the participant and his paired opponent were both identified and referred to by a specific participant number.

Basic intuition and a number of formal theories predict that incentives will positively affect performance [62]. Although these theories propose different mechanisms by which incentives affect performance, they all suggest that incentives increase the intensity and/or duration of effort, which in turn leads to increased performance [63]. Therefore, in our experiments the participants were incentivized by knowing that the winner would gain a material prize.

The procedure of the (competitive) game

At the beginning of Stage 1, the participants from the treatment groups (Winners/Losers) read that they were going to take part in a “Combo Skills Competitive Game”. These participants received a fictional number (e.g. "*Your number for this task is: Participant number 114*"), and were informed that they were randomly paired with another participant who would be their opponent for the game (e.g. "*Your opponent for this task is: Participant number 161*"). In addition, participants read that in each pair the one who acquired more points, based on correct answers in each of the three parts, would be declared the winner.

The participants from the control group read that they were going to take part in a “Combo Skills Game”. These participants completed exactly the same task as the participants from the treatment groups, without any elements of competition. I.e. Instead of being told that they were going to compete against another participant, they were asked to follow the instructions and to complete the whole game.

The task was as follows: Several squares with digits inside them were presented on-screen and participants were asked to answer questions regarding these shapes. The game included three parts. In Part 1 the participants were asked to choose the answer that correctly described the order of the colors. E.g., the correct answer to the following example is the first option:


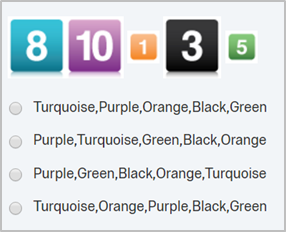


This part included six questions for which the participant was asked to choose the correct answer, each of which was presented on-screen for 10 seconds. Part 2 was similar to Part 1 but with a slight difference. In Part 2, the correct answer correctly described the order of the "Colors' Numerical Value," in descending order. The value of each color was determined by the number which was written on it. E.g., in the presented example the correct answer is the second option. As in Part 1, six questions were presented for 10 seconds each, for which the participants had to choose the correct answer. Part 3 was based on the same shapes as well, but in contrast to the two previous sections, this part included only one image, which was presented on-screen for 30 seconds. The participants were instructed to remember as many details as possible of the squares and digits that they saw on-screen, and informed that afterwards they would be asked to answer several questions regarding the shapes they had just seen. After the shapes were displayed for 30 seconds, the participants had 20 seconds to answer the following questions: (1) "Which digit appeared in the shape that was in the middle?" [10/1/5/8/3], (2) "Which digits were in large shapes?" [8,10,1/10,1,3/8,10,3/5,8,10], (3) "How many shapes appeared in the picture?" [5/7/9/11/13], and (4) "In which color was the lowest digit?" [Turquoise/ Purple/Orange/Black/Green]. Part 3 was purposefully designed to be very difficult to answer correctly since we wanted to raise the chances that even fast-thinking participants (who therefore believed themselves likely to win a competition based on their performance in the first two tasks) would believe notifications that they had lost the game.

In the treatment groups, after completing the game, participants randomly received a winning announcement (e.g. " *You won!!! You beat participant number 161! Due to your victory, you will participate in an Amazon Gift Card raffle*"), or a losing announcement (e.g. " *You lost... Participant number 161 beat you. Due to your loss, you will not participate in an Amazon Gift Card raffle*").

**References**

57. Garcia SM, Tor A, Gonzalez R. Ranks and rivals: a theory of competition. Personality and Social Psychology Bulletin. 2006 Jul;32(7):970–82. doi: 10.1177/0146167206287640

58. Garcia SM, Tor A, Schiff TM. The Psychology of competition: a social comparison perspective. Perspectives on Psychological Science. 2013;8(6):634–50.

59. Haran U, Ritov I. Know who you’re up against: counterpart identifiability enhances competitive behavior. Journal of Experimental Social Psychology. 2014 Sep;54:115–21. doi: 10.1016/j.jesp.2014.04.009

60. Shechter S, Hardisty D. Preferences for rank in competition: Is first-place seeking stronger than last-place aversion? Judgment and decision Making. 2020;15(2):246–

61. Ten Velden FS, Baas M, Shalvi S, Preenen PTY, De Dreu CKW. In competitive interaction displays of red increase actors’ competitive approach and perceivers’ withdrawal. Journal of Experimental Social Psychology. 2012 Sep;48(5):1205–8.

62. Bonner SE, Sprinkle GB. The effects of monetary incentives on effort and task performance: theories, evidence, and a framework for research. Accounting, Organizations and Society. 2002 May;27(4–5):303–45.

63. Hecht G, Tafkov I, Towry KL. Performance spillover in a multitask environment*. Contemporary Accounting Research. 2011 Nov 17;29(2):563–89. doi: 10.1111/j.1911-3846.2011.01114.x
